# Historical Review of the Fluid-Percussion TBI Model

**DOI:** 10.3389/fneur.2016.00217

**Published:** 2016-12-02

**Authors:** Bruce G. Lyeth

**Affiliations:** ^1^Department of Neurological Surgery, University of California Davis, Davis, CA, USA

**Keywords:** fluid percussion, traumatic brain injury, experimental brain injury, brain trauma, concussion

## Abstract

Traumatic brain injury (TBI) is a major health concern worldwide. Laboratory studies utilizing animal models of TBI are essential for addressing pathological mechanisms of brain injury and development of innovative treatments. Over the past 75 years, pioneering head injury researchers have devised and tested a number of fluid percussive methods to reproduce the concussive clinical syndrome in animals. The fluid-percussion brain injury technique has evolved from early investigations that applied a generalized loading of the brain to more recent computer-controlled systems. Of the many preclinical TBI models, the fluid-percussion technique is one of the most extensively characterized and widely used models. Some of the most important advances involved the development of the Stalhammer device to produce concussion in cats and the later characterization of this device for application in rodents. The goal of this historical review is to provide readers with an appreciation for the time and effort expended by the pioneering researchers who have led to today’s state of the art fluid-percussion animal models of TBI.

## Introduction and Purpose

Traumatic brain injury (TBI) is a major health concern worldwide (1, 2). In the United States, approximately 1.7 million people each year sustain a TBI, resulting in 275,000 hospitalizations and 52,000 deaths (3). Survivors of TBI often face an extended period of disruption of normal life functions affecting personal, family, and work situations. Severe TBI patients experience disabilities that often require extensive rehabilitation. There is an ongoing need to better understand the mechanistic underpinnings of TBI pathophysiology and develop treatments and interventions to aid TBI patients. Laboratory studies utilizing animal models of TBI are essential for addressing pathological mechanisms of brain injury and development of innovative treatments. Several preclinical TBI models are currently in use to address these critical needs. One of the most extensively characterized and widely used models is the fluid-percussion technique. There are several excellent reviews detailing the extensive list of scientific contributions utilizing the fluid-percussion technique that have contributed to our understanding of TBI mechanisms and development of experimental therapeutics (4, 5). In addition, several publications have described detailed practical techniques for performing fluid percussion in rodents (6–8). The purpose of this review is to provide an historical overview of the fluid-percussion model from its inception in larger animals to its current predominant use in rodents.

## Early Attempts at Modeling TBI in the Laboratory

Experimental models of TBI apply various forms of mechanical energy to the brain with a goal of replicating in animals the neurological syndromes, biological responses, and pathophysiology observed in human closed head TBI. Thus, animal models provide a means of rigorously studying clinical pathological states of TBI in a scientifically controlled manner in a laboratory setting. Over the years, animal models of TBI have employed numerous techniques applied to various species in attempts to reproduce multiple aspects of human TBI. Techniques include rapid acceleration or rotation of the head (9), impact to the freely moveable head (10–12), direct cortical impact to the dura mater and brain (13) and various fluid-percussion methods that rapidly inject a fluid into the closed cranium (14). The fluid-percussion technique has flourished and become one of the most widely used methods of producing TBI in the laboratory and has been applied to a number of species, including rabbit (15), dog and sheep (16), cat (17), pig (18, 19), and mouse (20), with the overwhelming majority of publications in rats (4).

## Emergence of Percussion Models

### Early Fluid-Percussion Models

The earliest application of a fluid-percussion technique involved striking a fluid-filled column secured to the exposed dura of an animal. Denny-Brown and Russell attempted to produce a generalized loading to the brain rather than a focal injury by applying a localized pressure pulse to the exposed dura of cats (21). They called this technique of rapidly applying an extradural injection of fluid a “percussion concussion” to distinguish the injury from an acceleration concussion. A decade later, Gurdjian et al. used a similar technique in mongrel dogs, applying compressed air in place of a fluid to transiently and rapidly raise intracranial pressure in an attempt to produce concussion (22). Later studies produced concussive brain injury by rapidly activating a plunger that injected fluid onto a column of water attached to the rabbit skull (23–25) or by dropping a weight onto a water column attached directly to the cerebral cortex of dogs (26). Stalhammar and colleagues in Sweden developed a more sophisticated device to control for different durations and amplitudes of a fluid pulse to the brain of rabbits through a 17.5-mm diameter midline craniectomy with dura removed (27–29). The device consisted of a 300-mm long Plexiglas cylinder filled with physiological saline and capped at one side by a piston that was struck by an adjustable mass (2–7 kg) suspended by a pendulum, whose fall height was also adjustable (0.2–0.5 m) (27). The device produced controlled, direct fluid/mechanical loading to the rabbit brain that Stalhammar described as the “contre-coup end of a combined cylinder-skull container” (28). The device produced pathophysiological changes in brain stem functions associated with concussion and was the direct predecessor to the present day Richmond fluid-percussion device first used in cat studies of concussion (described in Section “Early Years: Cat Central Fluid Percussion”). The modified fluid-percussion device was adopted by other early investigators to produce experimental TBI in a diversity of animal species, such as pigs (18), dogs, and sheep (16).

### Early Years: Cat Central Fluid Percussion

The Richmond group at the Medical College of Virginia, led by Becker, Povlishock, and Hayes further modified the Stalhammar device and applied fluid percussion to the midline of the cat brain through an 18-mm diameter craniectomy (14, 30, 31). A prolific number of publications by the Richmond group characterized the cat fluid-percussion model and provided a solid foundation for the study of mechanisms by which an impact to the head produced pathological responses in the brain. Thus, the early development of the cat fluid-percussion model provided neurosurgeons for the first time with a valid and reliable method for studying head injury in the laboratory. Of equal or greater importance, the cat fluid-percussion model opened the doors to the study of head injury by neuroscientists and the early development of the sub-discipline of Neurotrauma. The initial mechanistic studies using the Richmond cat fluid-percussion model focused on oxidative metabolism (32) and arachidonic acid metabolism (33) followed by investigations of the effects of naloxone in the study of endogenous opioids in hypotension after concussive brain injury (34). Further studies of endogenous opioids were carried out to examine their potential role in mediating secondary brain damage after TBI (35). A later series of studies, focusing on concussion, examined brain energy metabolites (36), regional rates of glucose utilization (37), and traumatic unconsciousness (38–40). Neurological function akin to the Glasgow Coma Score (41) was assessed (42), but little to no attention was placed upon functional behavioral outcomes, likely due to the great difficulty in performing behavioral testing in cats. Thus, the growing necessity to assess functional behavioral endpoints was the impetus for the subsequent development of the rat fluid-percussion model of TBI.

## Development of the Rat Fluid Percussion

### Rat Central/Midline Fluid Percussion

The next major advance in the fluid-percussion technique was Dixon’s development of midline fluid percussion in the rat. The impetus for developing a rat model of TBI was the goal to expand laboratory TBI model endpoints to behavioral assessment of outcome. The choice of the rat took advantage of the rich history in the field of psychology that made extensive use of behavioral analysis in this species. Dixon’s landmark study characterized the pathophysiological, histopathological, neurological, and behavioral responses to a range of injury severities in the rat (43). Dixon’s development of a rat neurological evaluation battery consisting of tests measuring acute reflex suppression analogous to the Glasgow Coma Scale also became a significant contribution to the field of experimental TBI (43). This neurological battery was based on earlier studies in cat fluid percussion by the Richmond group’s quantification of cat responsiveness to external stimuli and examination of the relationship between fluid-percussion injury severity and duration of behavioral suppression (39, 40). Dixon’s measures of sensorimotor behavioral responses to TBI were adapted from Feeney’s beam walk evaluations of rats with motor cortex ablations (44). McIntosh et al. performed a follow-up independent characterization of physiological, histopathological, and neurological responses to low and high magnitudes of midline fluid percussion in the rat, which demonstrated the rat model’s inter-laboratory reliability in reproducing pathological responses similar to those observed in human head injury (45). The rat afforded an ideal subject for evaluating neurological reflex responses (43, 46), and sensorimotor behavior (47) with later studies evaluating cognitive functions including learning and memory (48).

The development of the rat fluid-percussion model also afforded several other advantages including genetic homogeneity, ethical considerations of moving away from the use of companion animals to those lower on the phylogenetic scale, ease of procurement, and availability through a network of professional vendors. Furthermore, there were practical and financial advantages of using a species that required less vivarium space, afforded ease of handling, and reduced financial burdens of procurement and lower *per diem* costs of maintenance.

Perhaps the main advantage of moving to a rat model of TBI was to make feasible and practical, testing of dose–response pharmacological interventions that require behavioral assessment of large numbers of subjects. The first such dose–response pharmacological intervention study administered scopolamine to examine muscarinic cholinergic receptor involvement in transient behavioral suppression (traumatic unconsciousness) and physiological responses to TBI (46). That study was followed by an examination of muscarinic receptor involvement in longer term sensorimotor deficits associated with TBI (49). Taken together, this series of papers (characterizations of the rat midline fluid-percussion model and the application of pharmacological interventions) set the stage for a plethora of studies examining mechanisms of TBI pathology and development of preclinical experimental interventions. Thus, the rat fluid-percussion model made such studies not only possible but also practical and affordable.

### Rat Lateral Fluid Percussion

An extremely important alteration to the central rat fluid-percussion model, instituted by McIntosh, moved the craniectomy position from the vertex to a lateral (or more precisely, parasagittal) site (50). The intention for moving the craniectomy laterally was to generate in the rat fluid-percussion model, the coup-contrecoup injury commonly observed in human TBI (4). The McIntosh parasagittal orientation centered the craniectomy midway between Lambda and Bregma and midway between the sagittal suture and the lateral ridge at the intersection of the parietal and temporal bones. While the coup-contrecoup goal was not achieved, the lateral orientation model did reliably produce a mixed pathological model involving focal ipsilateral injury (cortical contusion as well as hippocampal and thalamic cell death) and diffuse pathological characteristics [subarachnoid hemorrhage, axonal injury, and neurochemical alterations (51–55)]. The focal contusion was not generated directly under the trephination site, but appeared considerably lateral to the actual site of the trephination, away from the direct fluid impingement onto the dura. The extent of histological pathology was associated with injury severity (51, 55, 56) and the contusion cavity progressively expanded for up to 1 year post-TBI (57, 58).

This innovative lateral rat fluid-percussion model provided several advantages over the central/midline model. First, rupture of the sagittal sinus underlying the central craniectomy, which could occur during surgery or upon percussive impact, was completely avoided, resulting in reduced blood loss and preservation of proper venous blood outflow from the brain. Second, the lateral model produced consistent cortical and hippocampal neuronal cell death compared to the central model. The gross pathology was always localized to the hemisphere ipsilateral to the fluid pulse, providing a relatively less injured, contralateral hemisphere useful for certain control comparisons. However, numerous studies have since demonstrated that the contralateral hemisphere sustains pathological neurochemical perturbations (52, 59) and diffuse white matter damage remote from the craniectomy site in rats and mice that render its use as a controlled comparison dubious (60).

Finally, the lateral fluid-percussion orientation appears to produce less direct brain stem compression allowing for application of higher fluid-percussion forces with lower mortality than central fluid percussion (50). Compared to the central/midline model, the lateral orientation model produces greater traumatically induced pathology in supratentorial brain structures. The lateral (parasagittal) approach has become the most commonly used orientation and remains one of the most utilized models in experimental TBI (4). The many studies that have utilized the lateral fluid-percussion rat model have established it as a valid and reliable model for studying the pathophysiology of human head injury (4). The lateral fluid-percussion model has led to the identification of cellular and molecular alterations caused by TBI and the subsequent development and evaluation of numerous experimental therapies.

The translation of experimental TBI therapies to successful positive human clinical trials has been overwhelmingly disappointing with responsibilities for these failures attributed to numerous inadequacies including insufficient characterization of therapeutic agents, limitations of animal models, and design flaws in clinical trials (61, 62). However, a recent severe TBI clinical study trial of amantadine, a pleotropic pharmaceutical with major actions of enhancing dopamine signaling as well as antagonist actions at the *N*-methyl-d-aspartate type glutamate receptor, demonstrated class I evidence (randomized controlled clinical trial) for improving disability rating scale scores in the chronic phase of TBI (63). Evaluation of amantadine in the rat lateral fluid-percussion model using a clinically relevant dosing paradigm based on the human trial reported improvements in cognitive function (64). The concordance of class I evidence demonstrating clinical benefit of amantadine in human TBI and the positive evidence in laboratory experiments provide pharmacological validation of the rat fluid-percussion injury model.

### Alterations to the Rat Lateral Fluid-Percussion Model

In most rat lateral fluid-percussion publications, the craniectomy was placed mid-distance between Bregma and Lambda and mid-distance between the sagittal suture and the lateral ridge (4.5 mm A–P and 3.0 mm M–L), as originally performed by McIntosh et al. (50). Over the years, laboratories have made alterations to these coordinates to account for pediatric versus adult rats and in attempts to alter the patterns of pathology of the adult rat. For example, the UCLA group moved the craniectomy site from McIntosh’s original location to a more lateral orientation located 6 mm away from the sagittal suture. They applied these coordinates to both pediatric rat studies (17–20 days old) (65) and adult rat TBI studies (66, 67). Two systematic studies reported that relatively small changes in craniectomy position (~1.0 mm shifts in location) produced significant alterations in histopathology and behavior (68, 69). Thus, precise and consistent placement of the fluid-percussion craniectomy is essential to ensure reproducibility of lesion parameters (size and precise location) and consistency in behavioral deficits within a study.

## Pig Fluid Percussion

While there are many benefits associated with the use of rodent models of TBI, there are also arguments for the use of higher order animals to better characterize the relevant pathobiology of human TBI. The use of multiple species is encouraged to address fully the complex pathobiological processes ongoing in human TBI (70). The lissencephalic nature of the rodent cortex has been recognized as a deficiency limiting the complete modeling of pathology occurring in the complex gyri and sulci of TBI patients (70). Armstead and Kurth initially adapted the fluid-percussion method to newborn piglets (1–5 days old) in studies of opioids in TBI (71) followed by a series of studies examining other effectors of cerebral hemodynamics in newborn and juvenile (3–4 weeks old) piglets (19, 72, 73).

A German group led by Bauer characterized a severe lateral fluid-percussion model over the right parietal cortex in juvenile pigs (6 weeks old mixed German domestic breed), which reliably produced secondary elevations in ICP accompanied by patterns of diffuse brain damage (74). More recently, a study of neuroinflammation in adult Yucatan micro pigs subjected to central fluid percussion found acute microglial process convergence on proximal axonal swellings suggesting its potential as a diagnostic and/or a therapeutic target (75). Pigs have become the higher order species of choice with the fluid-percussion technique.

## Mouse Fluid Percussion

The robust proliferation of genetically altered mice has provided opportunity to more precisely focus mechanistic studies on pathological processes. Following the lead of the Penn group who adapted the controlled cortical impact for mice (76), Carbonell and colleagues adapted the lateral fluid-percussion technique for mice with the craniectomy scaled down from the rat diameter of 4.8 to 2 mm and performed a neurological, behavioral, and histopathological characterization (20). The surgical preparation for mouse fluid percussion poses certain technical challenges, not unlike those encountered in pediatric rats. The surgical difficulty in drilling the fragile cranium with more flexible cranial sutures posed challenges that were met with alterations in the number and placement of anchor screws as well as the use of a gel form of cyanoacrylate adhesive to secure the injury cannula (20). In general, neurological and behavioral deficits as well as histopathological findings in mice were similar to those of the rat model, but with the temporal progression of neuronal injury proceeding more rapidly in mice (56). Later studies incorporated a larger diameter craniectomy (3 mm) in lateral (77–79) and central orientations (80, 81). The extensive availability of genetically altered mice and the development of fluid-percussion and other mouse models of TBI provides unique opportunities for investigators to more precisely explore pathological mechanisms of TBI.

## Variations in the Fluid-Percussion Device

Several different fluid-percussion devices have been developed since the inception of the Richmond device in the 1980s. The Dragonfly fluid-percussion device, used primarily by Shima’s group in Japan, applies the same basic principle of a closed hydraulic system as the Richmond device, but differs with smaller dimensions and stainless steel construction in place of Plexiglas™ (82, 83). Faden’s group devised a microprocessor-controlled, pneumatically driven instrument that addressed some of the concerns and limitation of the Richmond device (e.g., accurate leveling and removal of air bubbles) (6). Their device consists of a microprocessor-controlled assembly that initiates an impactor assembly with an air-driven impactor, producing a pressure wave in the form of a fluid bolus directly onto the exposed dura. Advantages of this device include precise control of the impact pressure and dwell time of the fluid pulse into the cranial vault (6). Pfister’s group recently developed a voice-coil linear motion actuator device for fluid percussion, which directly controls the motion of a piston of a hydraulic cylinder while utilizing a closed feedback system to generate fluid-percussion waveforms with adjustable rise times, peak pressures, and durations (84). These recent modifications of the fluid-percussion device are capable of providing greater control and flexibility of fluid pressure injury waveforms to the brain and provide greater flexibility in modeling different brain loading parameters.

## Consideration for Researchers

There are a number of important parameters to consider when utilizing the fluid-percussion technique regardless of the subject species. Craniectomy location (central versus lateral rat fluid percussion) produces unique pathological responses (45, 50). Relatively small alterations in the location of the craniectomy (~1.0 mm) also produce significant alterations in histopathology and behavior in rat lateral fluid percussion (68, 69). The magnitude of injury that is often categorized into a general trichotomy of mild, moderate, and severe can be foremost manipulated by altering the amount of energy applied to the device’s fluid-filled column or cylinder. Injury magnitude is often measured by histopathology, neurological and/or behavioral responses, as well as physiological responses such as blood pressure, EEG, or biomarkers. A prime example is the original rat fluid-percussion characterization paper in which the pendulum was released from different predetermined heights (thereby varying the potential energy), resulting in a range of pressures delivered to the brain and resultant range of pathological responses (43). Recent computer-controlled fluid-percussion devices can generate fluid-percussion waveforms with adjustable rise times, peak pressures, and durations all of which can alter the magnitude of injury [e.g., Ref. (84)]. While injury parameters such as peak pressure are manipulated to achieve a desired magnitude of injury, it is important to realize that that there is no standardization across laboratories. The accuracy of the precise pressure pulse delivered to the animal’s brain is affected by the location of the pressure transducer that is almost always located on the outlet of the fluid-percussion device and not within the actual cranium. Thus, the peak pressure measurement associated with a specific magnitude of injury may be different across laboratories. Furthermore, the length and compliance of tubing connecting the device (containing the pressure transducer) to the craniectomy can alter the pressures and the fluid dynamics of the pressure wave. Thus, the commonly reported fluid-percussion peak pressure should be treated as unique to that specific laboratory. Thus, it is essential to conduct pilot experiments to establish the fluid-percussion parameters necessary to achieve the desired pathological response in one’s own laboratory setting.

## Summary and Conclusion

From the early 1940s through the 1970s, pioneering head injury researchers developed and tested a number of fluid percussive methods to reproduce the concussive clinical syndrome in laboratory animals. By today’s standards, the work of these early pioneers in the field of Neurotrauma appear somewhat crude. However, it was their ingenuity and determination that laid the foundation for the level of sophistication found in today’s animal models of TBI using well-characterized and controlled devices and procedures such as fluid percussion and controlled cortical impact. The early fluid-percussion studies in cats provided a benchmark standard in the 1980s, producing groundbreaking studies of the mammalian response to brain trauma. The early cat fluid-percussion studies contributed greatly to our understanding of the brains response to head injury ranging from cerebral vascular alterations to traumatic disruption of consciousness. The development and characterization of rat fluid-percussion injury in the mid to late 1980s provided an important leap forward making possible behavioral outcome evaluations and a practical means of performing large scale studies of experimental therapeutics. Fluid percussion was later adapted for use in larger (pigs) and smaller animals (mice), providing means of studying TBI in gyrencephalic and genetically altered animals. The historical perspective of this brief review will hopefully provide appreciation for the effort, determination, and ingenuity expended by pioneering researchers who have led to today’s state of the art fluid-percussion animal models of TBI.

## Author Contributions

BL wrote and edited the manuscript.

## Conflict of Interest Statement

The author declares that the research was conducted in the absence of any commercial or financial relationships that could be construed as a potential conflict of interest.
